# The national essential medicines list of India: Time to revise and purge the mistakes

**DOI:** 10.4103/0976-500X.72346

**Published:** 2010

**Authors:** B Gitanjali

**Affiliations:** *Section Editor, JPP*

The World Health Organization (WHO) revises the model essential medicines list (EML) every two years. A visibly transparent and difficult process which is done by a team of experts who critically analyze the available evidence and give to the process their expertise and experience on evaluating published studies. Given the fact that the EML has been proven to improve availability, reduce prices, facilitate optimal use of drugs and improve safety,[1] it is surprising that the Government of India, in general and the Ministry of Health and Family Welfare, in particular, have not done anything to revise the list for the past seven years.

Considering the evidence that the EML has contributed significantly to improving access to medicines in many countries of the world, it is rather surprising that in our country no one seem to be enthusiastic to revise it. Some noises were made at the previous annual conference of the Indian Pharmacological Society held in Kolkata, where a meeting on the proposed revision was held, some timelines drawn, measures to ensure adequate representation from the different parts of the country were discussed, promises on the list being based on published evidence made and… nothing has happened for nine months. This is reflective of the apathy of the pharmacologists who obviously do not believe that revising the national EML (NEML) is worthy of their time and effort (unless it is given to them as gift authorship).

The commentary on the NEML 2003 by Manikandan S (page no. 75), is a chilling reminder of our lack of attention to detail, disregard for the exacting finer points of medicine and indifference to the potential harmful consequences the mistakes in the list could generate. In many countries all over the world, the EML is used as the basis for procurement of medicines. Organizations like the *Medicines Sans Frontiers* (MSF) use the model list of the WHO to base procurement for its causes all around the world. What gets into the list becomes a standard,[2] sometimes encouraging pharmaceutical companies to manufacture the formulation or strength of the medicine, knowing fully well that the demand will be there.

The question that perhaps will be on most readers’ minds is ‘so what if there are a few mistakes in the NEML? Surely no harm will come out of it?’ It is exactly this kind of attitude which endorses mediocrity and glorifies third rate productivity with a near total disregard for quality that has spawned the mess we are in, with reference to rational use of medicines. In many states of India, the NEML forms the backbone of the state EML. The mistakes that are found in the NEML will easily get incorporated into the state EML, because it is easier and quicker to clone the NEML as the state list. Procurement is also usually done by an agency not connected with the preparation of the list. Hence, what is on the list will automatically get into the procurement or tender list and the items so bought will reflect either the mistakes of the EML or will not get purchased because they will not satisfy the technical specifications. Therefore, patients, healthcare workers, administrators, government—everyone stands to lose. The EML is also used as a reimbursement list for insurance purposes and many a patient will be denied reimbursement of costs incurred. Formularies too echo the medicines in the NEML and so, the list of potential negative implications can go on and on.

Having said so, we should also introspect as to why it has taken seven years for someone to point out the numerous mistakes in the list. Is it because no one is using the NEML or implementing it that the mistakes have stayed hidden from public view for so long? Or is it because we, as a nation are used to sweeping uncomfortable truths under the carpet? The first question raises issues that would go against all the known evidence for implementing an EML. Is the NEML some sort of a product that is produced to comply with unrealistic standards and measurement tools being flaunted by the WHO? We have no data to substantiate this and the pitiable state of affairs with regard to access to essential medicines in the public health facilities of this country is evidence to the contrary.

So, why does this happen and what can be done to prevent it from happening again, when the EML gets revised? India is a large country with talented, skilled, resourceful persons spread all over. However, when it comes to scientific publications mandated by the government, there seems to be a dearth of talent and a shortage of wise men and women. And so, the same names, same people, same groups come together to write every guideline, prepare each list, formulate every curriculum and plan every policy related to health. The GOBSAT (good old boys sat around a table) approach is what is encouraged, endorsed and engraved in unseen letters of gold. Although these men and women have been doing a pretty good job in the past, they are now out of sync with the times, unable to cope with increasing demands of time, physical and mental stress, old age and lacking the skills necessary to take advantage of the vast amount of electronic resources available for activities of this kind. This has lead to the slow but serious blunting of standards and a lowering of the bar. This is why even though we were one of the first countries to propose a limited list of medicines and were proud torch bearers of many innovations in rational use of medicines such as the Hathi Committee Report, we are now lagging far behind some of the other countries within the southeast Asia region in almost every aspect, whether it is pharmacovigilance, antimicrobial resistance, drug information, ethical drug promotion, empowering consumers on rational drug use, etc. I hope that the future will see younger minds, newer approaches and a strong sense of seriousness associated with any kind of national level activity. Let us hope the next NEML, whenever it is prepared, will make us proud.
